# Number of Veterinarians in the Different European Countries

**Published:** 1897-12

**Authors:** 


### ABSTRACTS FROM FOREIGN JOURNALS.

### GERMAN.

UNDER THE DIRECTION OF J. PRESTON HOSKINS, PH.D.,
PRINCETON, N. J.

NUMBER OF VETERINARIANS IN THE DIFFERENT EUROPEAN COUNTRIES.—The Minister of Agriculture in Italy has published some interesting statistics in regard to this subject, from which Müller gives the following table in the *Archiv für wissenschaftliche und praktische Thierheilkunde*, vol. xxii., No. 6 :

| Countries. | Total No. of veterinarians. | *One* veterinarian to square kilometres. | *One* veterinarian to number of horses. | *One* veterinarian to number of cattle. |
|---|---|---|---|---|
| Belgium . . . . . . . . | 495 | 60 | 570 | 2,793 |
| Bulgaria . . . . . . . . | 48 | 2014 | 9038 | 36,874 |
| Denmark . . . . . . . . | 475 | 81 | 864 | 3,571 |
| Germany . . . . . . . . | 3516 | 153 | 1093 | 4,993 |
| France . . . . . . . . | 3389 | 129 | 1028 | 3,835 |
| Greece . . . . . . . . . | 17 | 3830 | | |
| Great Britain . . . . . | 2698 | 116 | 639 | 3,290 |
| Holland . . . . . . . . | 437 | 75 | 623 | 3,506 |
| Italy . . . . . . . . . | 2561 | 112 | 622 | 1,868 |
| Luxemburg . . . . . . | 23 | 112 | 719 | 3,459 |
| Norway . . . . . . . . | 123 | 2620 | 1227 | 8,153 |
| Austria . . . . . . . . | 957 | 312 | 1678 | 9,032 |
| Portugal . . . . . . . . | 88 | 1058 | 2263 | 7,931 |
| Roumania . . . . . . . | 133 | 985 | 4517 | 18,595 |
| Russia (excluding Poland) . | 853 | 5037 | 20961 | 28,427 |
| Russian Poland . . . . . | 72 | 1554 | . . . . | 38,188 |
| Sweden . . . . . . . . | 310 | 1422 | 1598 | 8,013 |
| Switzerland . . . . . . | 571 | 71 | 182 | 2,071 |
| Servia . . . . . . . . . | 45 | 1080 | 3666 | 18,394 |
| Spain . . . . . . . . . | 3432 | 146 | 599 | 646 |
| Hungary . . . . . . . . | 732 | 444 | 2388 | 6,655 |

—From *Tijdshrift voor Veeart seneikunde en Veeteelt*, vol. xxii., No. 2.

